# Optimization of pharmacotherapy in conjoined twins: a structured literature search with case applications and educational insights

**DOI:** 10.3389/fphar.2026.1709734

**Published:** 2026-03-30

**Authors:** Nadir Yalçın, Kaan Yılmaz, Hasan Tolga Çelik, Karel Allegaert

**Affiliations:** 1 Department of Clinical Pharmacy, Faculty of Pharmacy, Hacettepe University, Ankara, Türkiye; 2 Faculty of Pharmacy, Hacettepe University, Ankara, Türkiye; 3 Division of Neonatology, Department of Pediatrics, Faculty of Medicine, Hacettepe University, Ankara, Türkiye; 4 Clinical Pharmacology and Pharmacotherapy, Department of Pharmaceutical and Pharmacological Sciences, KU Leuven, Leuven, Belgium; 5 Child and Youth Institute, KU Leuven, Leuven, Belgium; 6 Department of Development and Regeneration, KU Leuven, Leuven, Belgium; 7 Department of Hospital Pharmacy, Erasmus MC, Rotterdam, Netherlands

**Keywords:** conjoined twins, cross-circulation, drug dosing, medication optimization, neonatal care, pharmacokinetics

## Abstract

**Introduction:**

Conjoined twins present a rare but clinically challenging scenario requiring highly individualized pharmacologic strategies. Anatomical fusion, shared circulatory systems, and organ overlaps complicate drug absorption, distribution, metabolism, and excretion, including the neonatal intensive care unit setting and perioperative care. This review aims to synthesize current evidence and clinical experience regarding pharmacotherapy in CTs, focusing on drug selection, therapeutic drug monitoring and individualized dosing strategies based on anatomical and physiological variations.

**Methods:**

We performed a comprehensive structured literature review using PubMed database, covering the period 1970–2025, restricted to English-language publications. Case reports and reviews relevant to pharmacology in CTs were included (n = 4/93); surgical, radiological or anesthetic-only reports without pharmacologic content were excluded. We integrated these findings with two case reports involving pygopagus and thoracopagus conjoined twins treated in our tertiary referral care hospital. Key pharmacokinetic variables such as volume of distribution, renal clearance, and enteral absorption were examined in relation to the cross-circulation status. Additionally, an online quiz was conducted among clinicians to assess baseline knowledge.

**Results:**

Our results observations suggest that drugs such as amikacin require TDM-based adjustments in the presence of cross-circulation in both subjects. Shared renal or gastrointestinal anatomy further necessitates titrated and monitored dosing regimens. Emergency medication strategies should consider whether complete, partial, or absent circulatory sharing is present. Questionnaire data revealed unexpectedly high knowledge levels among physicians and pharmacists, though further educational enhancements—such as virtual reality simulation and tailored protocols—are recommended.

**Discussion:**

Pharmacologic management in CTs demands a multidisciplinary approach, close monitoring, and careful documentation. Case-based strategies and educational reinforcement can reduce risk and improve outcomes. Further research, including the establishment of central registries and the use of physiologically based pharmacokinetic modeling, is essential to inform individualized care in this very rare population.

## 1 Introduction

Conjoined twins (CTs), or Siamese twins, are monozygotic twins whose embryonic division is incomplete, leading to partial anatomical fusion. This condition, affecting approximately 1 in 100,000 live births, is associated with high morbidity and mortality rates (Savulescu and Persson, 2016; Kusmayadi et al., 2024). Anatomical fusion types, particularly thoracopagus and craniopagus, critically affect the feasibility of medical and surgical interventions (O'Connell, 1976; Heuer et al., 2019). The etiology of conjoined twinning remains uncertain and is generally attributed to incomplete embryonic division or secondary fusion during early development. Epidemiological data suggest a higher reported incidence among female fetuses and a predominance of thoraco-omphalopagus configuration. No consistent hereditary pattern or clearly established maternal risk factors have been identified (Mackenzie et al., 2002; Kusmayadi et al., 2024).

Among the most complex aspects of conjoined twin care is pharmacotherapy. Shared circulatory systems (shunts), organ overlaps, and divergent metabolic functions necessitate nuanced, often case-specific pharmacological strategies (Kusmayadi et al., 2024). CTs exhibit varying degrees of organ sharing. Thoracopagus twins are joined at the chest and frequently share cardiac structures; omphalopagus twins are connected at the anterior abdominal wall, often sharing the liver; pygopagus twins are fused at the sacral or gluteal region; ischiopagus twins are joined at the pelvis with variable sharing of lower abdominal and pelvic organs; and craniopagus twins are fused at the skull with potential sharing of cerebral vasculature. Cross-circulation is extensive in thoracopagus and craniopagus, variable in omphalopagus, and minimal in pygopagus or ischiopagus (Singla et al., 2024). Thoraco-omphalopagus twins, the most common type, often share the liver and gastrointestinal structures (Bindlish and Sawal, 2022). Scintiangiography and MRI can delineate parenchymal fusion and functional dominance (Margouleff et al., 1980). Vascular anatomy and shunts determine the extent of cross-circulation—a key factor in drug pharmacokinetics. Early diagnosis through fetal MRI and Doppler ultrasound aids in pre-delivery planning, particularly for anesthetic and antimicrobial protocols (Chalam, 2009; Patil et al., 2016; Frawley, 2020). At the risk of oversimplification, a conjoined twin can be considered as a two-compartment system, with variability in intercompartmental flow (Q).

### 1.1 Absorption

Gastrointestinal malformations in conjoined twins, particularly those with thoraco-omphalopagus configuration, have significant implications for both drug absorption and systemic distribution. Shared or fused gastrointestinal tracts—especially in the region of the stomach or duodenum—can result in unpredictable enteral absorption kinetics. Feeding regimens (continuous vs. bolus) and enteral tolerance further modify absorption, as in NICU practice for preterm neonates (Frawley, 2020). When enteral medications are administered to one twin, the extent to which the drug is absorbed, metabolized, or redirected through shared vascular pathways becomes highly variable. Additionally, the presence of anatomic fusion or duplication of the intestines and uncertainty in splanchnic circulation may lead to asymmetric or delayed drug absorption between the twins, complicating dosing strategies and therapeutic monitoring (Boles and Vassy, 1979).

Many thoracopagus twins exhibit varying degrees of gastrointestinal fusion, particularly around the duodenum where most enteral drugs are absorbed. A key clinical approach involves using a trial dose of an enteral medication with observable effects (e.g., diuretics such as furosemide). In one case, a shared enteral dose led to modest diuresis, whereas individualized dosing based on twin-specific weight resulted in improved efficacy (Rodman and Placencia, 2018).

Moreover, shared intestinal vasculature may increase systemic exposure if enhanced perfusion facilitates drug absorption; conversely, asymmetric or compromised splanchnic blood flow may delay or reduce absorption, resulting in unpredictable bioavailability. For this reason, imaging-based assessments (e.g., contrast fluoroscopy or computed tomography angiography) may be warranted prior to initiating long-term enteral treatment regimens (Patil et al., 2016).

### 1.2 Distribution

**FIGURE 1:**
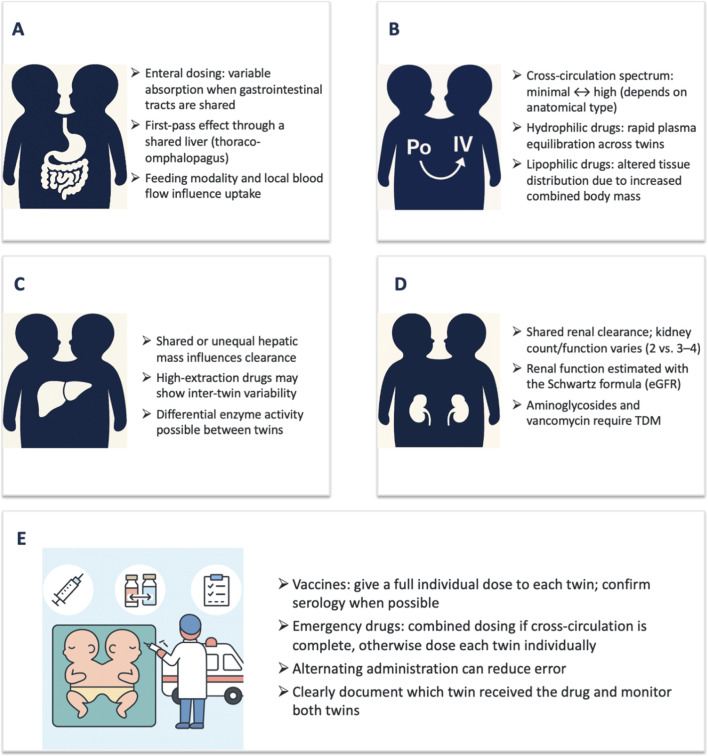
ADME considerations, transport and emergency care in neonates with conjoined twinning. Clinical management depends on anatomical type and degree of cross-circulation. TDM, imaging, and multidisciplinary planning are essential for safe pharmacotherapy. In Panel B, PO and IV symbols represent systemic drug exposure patterns after different routes of administration in the context of cross-circulation rather than absorption mechanisms. In Panel E, vaccines are included to illustrate practical considerations in individualized drug administration and monitoring in conjoined twins. **(A)** Absorption. **(B)** Distribution. **(C)** Metabolism. **(D)** Excretion. **(E)** Transport & Emergency care.

Volume of distribution (Vd) in conjoined twins depends heavily on cross-circulation and whether a medication is hydrophilic or lipophilic. Extensive cross-circulation may increase the effective volume of distribution for hydrophilic drugs by expanding the functional circulating plasma compartment across both twins. Studies recommend administering a test dose of such drugs to one twin and monitoring serum levels in both to assess shared blood volume (Rodman and Placencia, 2018). In one reported case, nearly identical gentamicin serum levels confirmed rapid and complete cross-circulation, justifying combined body weight-based dosing for certain scenarios (Rodman and Placencia, 2018; Frawley, 2020). For this reason, TDM is essential, particularly given neonatal variability in extracellular water and organ perfusion (Kearns et al., 2003; Rodman and Placencia, 2018) (Figure 1).

Lipophilic medications, which distribute into tissue rather than plasma, may require adjustment based on organ mass. Since conjoined twins may have augmented combined organ volume, lipophilic agents like ketamine or propofol may require cautious titration (Shank et al., 2005; Chalam, 2009). Determining the extent of cross-circulation can involve radionuclide imaging using 99 mTc-labeled radiotracers, simultaneous blood chemistry panels, and pharmacokinetic sampling (Chalam, 2009). In contemporary practice, advanced high-resolution MRI, 3D reconstruction, and virtual surgical planning techniques have further refined anatomical and vascular assessment in conjoined twins, although the fundamental principles of cross-circulation evaluation remain similar to earlier reports (Mohammad et al., 2023).

In addition to volume of distribution, drug distribution in conjoined twins may be influenced by altered plasma protein binding, particularly in neonates with physiologically reduced albumin and α1-acid glycoprotein concentrations. An increased free drug fraction may enhance pharmacologic effects as well as toxicity risk. Furthermore, shared plasma volume and variable inter-twin blood flow may lead to dynamic equilibration of circulating drug concentrations, complicating prediction of tissue exposure.

### 1.3 Elimination

Hepatic drug clearance may be further complicated by unequal liver mass distribution in xiphopagus conjoined twins (twins fused at the lower sternum/xiphoid region), especially when one twin contributes disproportionately to the shared hepatic parenchyma. This asymmetry can lead to inter-twin differences in drug metabolism and bioavailability, particularly for medications with a high hepatic extraction ratio. Scintiangiography using radiotracers such as 99 mTc–sulfur colloid has proven effective in preoperatively delineating liver fusion points and assessing functional hepatic contribution, offering a valuable tool for predicting pharmacokinetic variability (Margouleff et al., 1980).

Renal clearance represents another complex domain. Though conjoined twins may have fewer than four kidneys, compensatory hyperplasia is thought to enable sufficient filtration. In cases involving renally cleared antibiotics such as vancomycin or gentamicin, therapeutic drug monitoring (TDM) indicated effective clearance despite renal sharing (Rodman and Placencia, 2018). Shared renal function may either decrease individual drug clearance (if functional nephron mass is reduced) or maintain near-normal clearance through compensatory hyperfiltration, leading to variable elimination kinetics.

Case studies show that creatinine clearance can be similar to that of singleton neonates, provided kidney function is symmetrical and unobstructed. This is particularly important when nephrotoxic agents are employed. If only two kidneys are present, regular serum creatinine and drug trough levels are essential using neonatal formulas (e.g., Schwartz equation) (Schwartz and Work, 2009; Frawley, 2020).

### 1.4 Emergency medication and medication safety

Code events necessitate rapid decisions with minimal error tolerance. For twins with confirmed complete cross-circulation, one effective strategy includes alternating administration between the twins using combined weight-based dosages, minimizing preparation time and ensuring simultaneous drug effect (Sager et al., 2018). When cross-circulation is incomplete or unknown, dual individual dosing is recommended. For example, administering epinephrine to both twins at weight-appropriate doses avoids the risk of underdosing if the drug does not adequately distribute between them (Parmekar et al., 2018).

To further reduce medication-related errors, color-coding IV lines, pumps, and medications—coupled with consistent twin positioning—enhances accuracy during high-stress situations (Rodman and Placencia, 2018). Simulation-based training reinforces protocol adherence and team readiness (Sager et al., 2018). In the electronic medical record (EMR), documentation must reflect either half of the combined body weight per twin or clearly specify when medications are administered using a unified dosing strategy. In such cases, annotations must clarify which twin received the dose and the anticipated systemic exposure in both, preventing duplication or omission in care documentation (Luton et al., 2018; Greco et al., 2021).

### 1.5 Vaccination considerations

Due to shared circulation and increased volume of distribution, it remains uncertain whether single or double vaccine doses should be administered. In practice, individual full doses are recommended to ensure adequate immune responses, given variability in T-cell activity and antigen presentation. Immunization practices remain under-documented in CTs. Given potential under-response to vaccines, dual-dosing per twin, with post-vaccination serology, is currently the most cautious approach (Bindlish and Sawal, 2022).

This manuscript comprises three complementary components: (1) a structured literature review on pharmacologic management in conjoined twins, (2) a case series illustrating individualized pharmacotherapeutic decision-making in two clinical scenarios, and (3) an educational questionnaire assessing clinician awareness and knowledge related to these challenges. These components are presented sequentially to integrate available evidence, clinical application, and educational implications.

This review synthesizes findings from a comprehensive structured search on drug management in CTs and integrates recent publications (1970-2025) to develop actionable pharmacologic strategies, particularly concerning cross-circulation and drug distribution (2.). It also includes the medication management processes conducted by a clinical pharmacist in two distinct conjoined twin cases referred to our tertiary referral children hospital (3., along with the results of an online questionnaire with multiple choice administered to clinicians including physicians and pharmacists, to assess their knowledge and awareness related to these interventions (4.).

## 2 Literature search strategy

A structured literature search was conducted in PubMed/MEDLINE (17 September 2025) to identify publications addressing pharmacological management in conjoined twins. PubMed was selected as the primary database because it provides extensive coverage of biomedical literature and indexes the majority of peer-reviewed clinical case reports and pharmacology-related publications relevant to neonatal medicine and rare congenital conditions. In this field, the available evidence is largely limited to case reports and narrative reviews, which are predominantly indexed in MEDLINE. Nevertheless, we acknowledge that searching additional databases such as Embase could potentially increase retrieval sensitivity. Therefore, the use of a single database is recognized as a limitation of this review. The search strategy was developed using a combination of controlled vocabulary (Medical Subject Headings, MeSH) and free-text keywords in order to maximize sensitivity and capture both indexed and non-indexed articles. The following search string was applied.

(“Conjoined Twins”[Mesh] OR “Siamese Twins”[tiab] OR “conjoined twin”[tiab] OR “conjoined twins”[tiab] OR “Siamese twin”[tiab] OR “Siamese twins”[tiab]) AND (“Drug Therapy”[Mesh] OR “Pharmacology”[Mesh] OR “Pharmacokinetics”[Mesh] OR “Drug Administration Routes”[Mesh] OR “Drug Dosage Calculations”[Mesh] OR “Therapeutics”[Mesh] OR “drug therapy”[tiab] OR “drug treatment”[tiab] OR “pharmacologic”[tiab] OR “pharmacological”[tiab] OR “pharmacotherapy”[tiab] OR “pharmacokinetics”[tiab] OR “pharmacodynamic*”[tiab] OR “medication”[tiab] OR “medications”[tiab] OR “dosing”[tiab] OR “dose”[tiab] OR “dosage”[tiab] OR “therapeutic drug monitoring”[tiab] OR “TDM”[tiab])

To ensure clinical relevance, the search was restricted to studies involving human, pediatric populations (infants, newborns, children, and adolescents). In addition, publication type filters were applied to include case reports and review articles, which represent the predominant forms of evidence available for this rare condition. The search was limited to articles published in English. No restrictions were placed on publication year.

This strategy was designed to retrieve the broadest possible range of studies describing drug therapy, pharmacokinetics, pharmacodynamics, dosing, and therapeutic drug monitoring in conjoined twins. The retrieved records were subsequently screened according to PRISMA guidelines, and the selection process was documented in a flow diagram.

**FIGURE 2:**
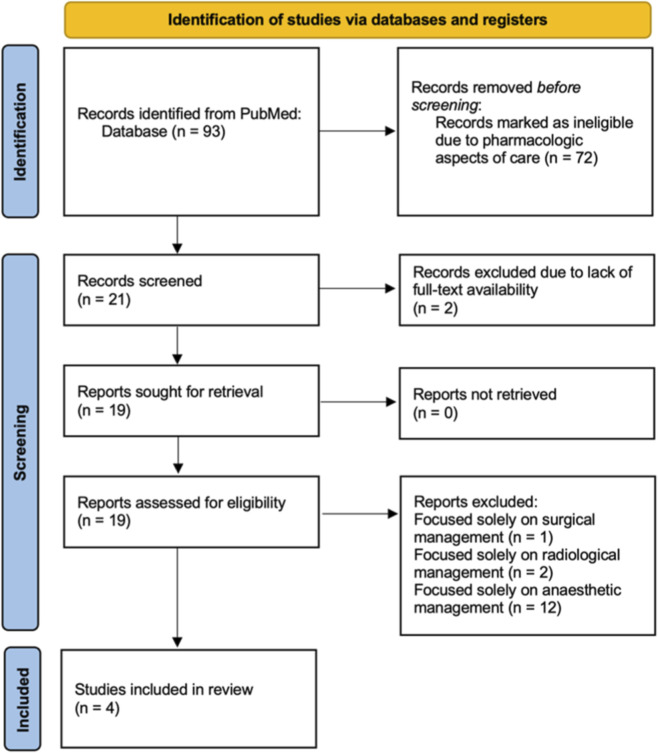
PRISMA flow diagram illustrating the study selection process for the comprehensive literature review.

The initial PubMed/MEDLINE search yielded 93 records (1970-2025), and the titles and abstracts of these were screened for eligibility by two reviewers (K.Y. and N.Y.). Disagreements regarding inclusion or exclusion were resolved through mutual discussion, and in cases where consensus could not be reached, the opinion of a third researcher (K.A.) was sought. Of these, 72 records were excluded because they did not pertain to conjoined twins or did not address pharmacologic aspects of care. The full texts of 19 potentially relevant and available articles were then assessed in detail. Following full-text evaluation, 15 articles were excluded for reasons such as lacking pharmacological data, focusing solely on surgical, radiological or anesthetic management without drug therapy details. Ultimately, 4 studies (two reviews and two case reports) met the predefined inclusion criteria and were included in this comprehensive literature review (Figure 2).

Data extraction was performed using a predefined standardized form. For each included study, the following information was recorded: year of publication and study type (case report or review), configuration type (e.g., thoracopagus, pygopagus), shared organ systems and cross-circulation status, drug(s) studied, dosage strategy and route of administration, therapeutic drug monitoring (TDM) practices, reported pharmacokinetic/pharmacodynamic observations, and clinical outcomes including safety data.

As all included publications were case reports or narrative reviews, conventional risk of bias tools designed for randomized controlled trials (e.g., Cochrane RoB) were not applicable. Instead, methodological quality and reporting adequacy were qualitatively assessed based on the clarity of clinical and anatomical descriptions, specification of drug dosing strategies, reporting of pharmacokinetic data (e.g., serum concentrations), and transparent presentation of outcomes and adverse effects.

The following section summarizes the pharmacologic findings derived from the four studies included in the structured literature review. One case report has noted that the liver in neonates has not yet developed fully effective mechanisms for drug metabolism, and that the low concentrations of drug-binding proteins (such as albumin and α1-acid glycoprotein) predispose patients to a higher proportion of unbound drug (Hobaika et al., 2010). Another review has noted that resuscitation drugs are administered at a dose determined by half of the twins’ combined weight, and that cross-circulation between conjoined twins has significant implications for anesthetic pharmacology (Frawley, 2020). In one reported case, all anesthesia equipment, medications, and blood products were duplicated and color-coded, with two distinct teams managing anesthesia simultaneously to minimize the risk of error (Kaddoum et al., 2024). Finally, the review by Rodman and Placencia provides the most comprehensive overview to date, emphasizing the critical role of cross-circulation in dosing decisions, differences in drug distribution between hydrophilic and lipophilic agents, and safety strategies such as color-coding and individualized identification to reduce medication errors; additional focus is given to uncertainties in enteral absorption and renal clearance (Rodman and Placencia, 2018).

## 3 Case series

### 3.1 Case twins 1

Female pygopagus conjoined twins (designated Twin A and Twin B), diagnosed prenatally, were born at 36^1/7^ weeks of gestation *via* cesarean section in Syria, weighing a total of 3950 g, as the first live birth of a 15-year-old primigravida. The twins were conjoined at the lower torso and pelvic region and were immediately transferred to the neonatal intensive care unit (NICU). The APGAR scores were recorded as 6/7/8.

Each twin possessed one functional kidney, and their uteri, urinary bladders, and urethrae were anatomically distinct. Gastrointestinal tracts were separate up to the distal end, with a common anal canal merging approximately 1 cm proximal to the anus. Additionally, neural tissue fusion was identified in the sacral region.

Given the presence of febrile values, empirical antimicrobial therapy was initiated to prevent early-onset neonatal sepsis. The regimen included vancomycin, amikacin, meropenem, and fluconazole. Considering the presence of a single kidney in each twin and to minimize the risk of nephrotoxicity, the clinical pharmacist recommended adjusting the amikacin dosing strategy. Instead of administering a full dose of 15 mg/kg/day based on total body weight through the vascular access of Twin B, the daily dose was divided into two equal parts and administered to both twins, with routine TDM of serum levels.

Amikacin was initially administered exclusively to Twin B at a dose of 15 mg/kg/day, calculated according to total body weight. On postnatal day 60 (corresponding to day 10 of therapy), trough concentrations were measured *via* a central venous catheter immediately prior to the next scheduled 24-h dose. The predefined therapeutic targets for neonatal amikacin therapy were peak concentrations of 20–30 mg/L and trough concentrations <5 mg/L to minimize nephrotoxicity. Unexpectedly, trough levels were markedly elevated in Twin B (13.1 mg/L) and were also elevated in Twin A (7.7 mg/L), despite Twin A not directly receiving amikacin. These findings suggested significant cross-circulation between the twins. Following clinical pharmacy consultation, the total calculated daily dose was divided equally between both twins. Repeat therapeutic drug monitoring on day 15 demonstrated trough concentrations within the target range (2.7 mg/L in Twin B and 4.0 mg/L in Twin A).

Ultimately, antimicrobial therapy was successfully administered within the target TDM range without inducing nephrotoxicity. Following stabilization, a tissue expander was implanted, and surgical separation of the twins was successfully performed.

### 3.2 Case twins 2

Female thoracopagus conjoined twins (designated Twin X and Twin Y), diagnosed prenatally, were born at 35^4/7^ weeks of gestation *via* cesarean section in Syria, weighing a total of 4030 g, as the first live birth of a 25-year-old multipara. The patients, who were intubated and in poor general condition, were immediately transferred to the NICU.

Abdominal ultrasonography revealed a single liver and gallbladder, with both kidneys visualized bilaterally. The urinary bladder was identified in Twin X but could not be clearly assessed in Twin Y. A single heart with shared major vascular structures was observed. Bedside echocardiography further demonstrated complex congenital heart disease characterized by a common atrium, septal defects, and functional single ventricle physiology. Pulmonary artery Doppler showed moderate gradients on the left (25 mmHg) and higher systolic gradients on the right (55 mmHg), indicating significant hemodynamic asymmetry.

In Twin Y, who exhibited hypertensive values due to a single shared vein and moderate-to-severe pulmonary stenosis, antihypertensive therapy could not be initiated, as it would potentially compromise Twin X’s cardiac function. At that time, both twins had tracheostomies and were receiving mechanical ventilatory support without inotropic therapy. Twin Y demonstrated blood pressure values ranging between 108 and 121/76–78 mmHg. Oxygen saturation levels were maintained between 75% and 80% under controlled ventilation. Given the presence of shared cardiac physiology and cross-circulation, pharmacologic reduction of systemic vascular resistance in Twin Y was considered likely to adversely affect systemic perfusion in Twin X. Therefore, antihypertensive therapy was withheld in favor of close hemodynamic monitoring. Hepatitis B vaccine was given as a single combined dose due to assumed cross-circulation. Also, due to elevated procalcitonin levels, empirical antimicrobial therapy including vancomycin, amikacin, meropenem, and fluconazole was initiated. Given the presence of shared circulation, TDM was applied specifically for amikacin. The blood samples were obtained *via* a central venous catheter on postnatal day 101, corresponding to day 3 of amikacin therapy. To assess the extent of inter-twin blood exchange, amikacin was administered to only Twin Y at a dose of 15 mg/kg based on total infant’s weight. Thirty minutes after the expected peak sampling time following the completion of the amikacin infusion, serum amikacin concentrations were simultaneously measured in both Twin X and Twin Y. Although Twin Y’s serum concentration was within the target range at 5.3 mg/L, the unexpectedly elevated level in Twin X, measured at 10.9 mg/L, highlights the critical importance of vigilant TDM in conjoined twins with shared circulation. The twins, who were tracheostomized and deemed inoperable for surgical separation, were declared exitus on day 148 of hospitalization despite all medical interventions.

Given the two cases presented here, statistical inference is severely limited and quantitative conclusions are not generalizable. The fact that both cases originated from the same geographic region likely reflects referral patterns and the availability of specialized care centers rather than true epidemiological clustering. Nonetheless, genetic and environmental factors, including the higher prevalence of consanguinity in certain regions, may contribute to the occurrence of complex congenital anomalies, although causality cannot be established in the present report. Case selection bias is also possible, as most pharmacologic data on conjoined twins are derived from high-volume surgical centers. Furthermore, cross-cultural genetic variability in drug metabolism remains largely unknown. Additionally, reporting bias must be considered, as published case reports may preferentially describe unusual, clinically significant, or unexpected pharmacokinetic findings, potentially limiting the generalizability of the available evidence.

## 4 Questionnaire

Prior to the online seminar titled 'Drug Optimization in Conjoined Twins' conducted by author K.A. and attended by physicians (n = 5) and pharmacists (n = 6), a brief multiple-choice quiz consisting of six topic-related questions was administered to the participants. All attendees were employed in the same hospital responsible for the care of the cases of conjoined twins reported. The results of the quiz indicated that the knowledge level of healthcare professionals regarding this rare clinical scenario was above expectations (Table 1). The high rate of correct responses to questions that were designed to integrate both theoretical and practical aspects suggests that individualized pharmacological management through a multidisciplinary team approach is feasible in such complex cases. These findings underscore the need for clinicians who are both experienced and specialized in managing conjoined twin cases to prevent drug-related complications and ensure optimal therapeutic outcomes. However, the small sample size (n = 11), the inclusion of only respondents from a single hospital with direct conjoined twin exposure, and the absence of a comparator group from institutions without such experience collectively limit the generalizability of the findings and introduce potential response bias.

**TABLE 1:** Correct response rates of the participants with questions and answers.

Questions and answers	Correct response rate, n (%)	Incorrect response rate, n (%)
1. Which of the following anatomical types of conjoined twins is the most common? a) Craniophagus ***b) Thoracopagus*** c) Ischiopagus d) Pygophagus e) Parapagus	5 (62.5%)	3 (37.5%)
2. What is the primary reason for individualizing drug dosage in conjoined twins? ***a) Shared organs and circulation can alter pharmacokinetics*** b) Drug metabolism is always identical in both twins c) They often suffer from kidney failure d) They are typically resistant to most medications e) Vaccines are always ineffective in them	7 (77.8%)	2 (22.2%)
3. What should be done if conjoined twins have a high degree of cross-circulation? a) Discontinue all medications b) Administer drugs to only one twin ***c) Titrate drugs gradually while monitoring both twins’ responses*** d) Use only topical medications e) Avoid any surgical intervention	11 (100%)	-
4. What is the primary risk when administering medication to one twin in a pair with significant cross-circulation? a) Rapid hepatic metabolism b) Ineffective absorption in the GI tract c) Drug elimination delays ***d) Unpredictable drug exposure in the other twin*** e) Resistance to antibiotics	9 (90%)	1 (10%)
5. Which pharmacokinetic parameter is most directly affected by cross-circulation in conjoined twins? a) Protein binding b) Half-life c) Receptor sensitivity d) Drug solubility ***e) Volume of distribution***	8 (72.7%)	3 (27.3%)
6. In conjoined twins with minimal or no cross-circulation, what is the most appropriate strategy for drug dosing? a) Administer half the dose to one twin only b) Apply topical medications to avoid systemic effects ***c) Dose each twin individually based on their body weight*** d) Use the same dose for both regardless of physiology e) Avoid all systemic medications	11 (100%)	-

## 5 Discussion

This review demonstrates that only a limited number of reviews and case reports have focused specifically on individualized pharmacotherapy in conjoined twins. Given the rarity of these cases, the available evidence remains sparse and largely descriptive. There is a clear need for further studies emphasizing TDM and optimal pharmacotherapy, ideally conducted within a multidisciplinary framework not only for conjoined twins but also for singleton neonates.

This paper also highlights the pharmaceutical complexity involved in treating conjoined twins. From of pharmacokinetics approach, a conjoined twin can be considered as a two-compartment system, with variability in intercompartmental flow (Q). Consequently, each case demands individualized planning based on circulatory, renal, and gastrointestinal anatomy. A consistent theme is the necessity for pre-treatment simulations, close monitoring, and interdisciplinary collaboration. The routine use of color coding, clear institutional protocols, alternating administration, EMR safeguards, and TDM contributes to improved clinical outcomes.

As illustrated with our case reports, individualized pharmacotherapeutic approaches are essential, taking into account both the pharmacological mechanisms of each drug and the unique anatomical features of conjoined twins. Particularly in the neonatal population - where physiological parameters are highly variable, organ systems are immature, and prolonged, delicate interventions are often required in the NICU - the importance of TDM and the timely selection of the right drug for the right patient becomes increasingly evident in the management of very rare and complex cases. Consistent with the literature(Rodman and Placencia, 2018), our cases also demonstrated that, as with gentamicin and vancomycin, TDM and treatment management strategies for amikacin may vary depending on the extent of shared (cross) circulation between conjoined twins.

The brief quiz conducted demonstrated that physicians and pharmacists possess an adequate level of knowledge regarding the pharmacokinetics of drugs in conjoined twins. However, to further enhance both theoretical and practical competencies, there is a clear need within healthcare education—including medicine, nursing, and pharmacy—for the integration of case-based simulations using virtual reality (VR) technology, 3D imaging, the development of specialized drug formulations, the expansion of medication administration processes, and the establishment of hospital-wide protocols tailored to such rare and complex clinical scenarios (Arnold et al., 2018; Juhnke et al., 2019; Shafarenko et al., 2022).

Conjoined twins must be individualized based on both the pharmacologic properties of the drug and the degree of shared circulation. In conjoined twins, drugs are generally administered *via* intravenous injection, as absorption following intramuscular administration is highly variable and often unpredictable (Donaldson et al., 1990). Hydrophilic intravenous medications may require single or split dosing depending on whether circulation is complete or separate. For lipophilic drugs and enteral formulations, considerations include shared organ systems and absorption variability. Emergency medications should be administered cautiously, with dosing tailored to circulation status to prevent under- or overdosing (Rodman and Placencia, 2018).

Conjoined twins present unique challenges requiring tailored approaches. Strategies include individual dosing for each twin, which ensures accuracy but may be time-consuming, and administering a combined dose to one twin, which simplifies calculations but carries a risk of dosing errors due to uncertain volume of distribution. Alternating combined-dose administration may be effective in cases with confirmed cross-circulation, offering both efficiency and better pharmacokinetic coverage (Rodman and Placencia, 2018).

Ethical challenges in pharmacotherapy for conjoined twins are profound. Parental consent is often obtained under highly stressful circumstances, where decisions regarding experimental dosing must be made with little or no precedent. The risk–benefit balance is further complicated by uncertainty, as one twin may be exposed to toxicity while the other remains undertreated. Clinicians carry a professional responsibility to document their rationale transparently, communicate uncertainties to families, and refrain from untested interventions outside compassionate or lifesaving contexts. A further dilemma lies in shared physiology *versus* individual rights: should drug dosing be determined for the pair collectively or for each twin separately? Core bioethical principles—autonomy, beneficence, non-maleficence, and justice—demand that each twin be considered individually despite anatomical fusion. In this context, the principle of clinical equipoise is particularly relevant, as any experimental dosing must be justified by genuine uncertainty regarding risk and benefit (Rojas, 2015). Moreover, ethical care extends beyond acute management: long-term follow-up is essential to capture developmental pharmacology, late toxicities, and the evolving health needs of each twin after separation or throughout life (Afzal and Montero, 2025). Finally, global collaboration is ethically imperative; the establishment of international registries could reduce the experimental burden on future cases by ensuring that subsequent patients benefit from accumulated evidence. (Savulescu and Persson, 2016).

While literature remains limited, current evidence supports a cautious approach—emphasizing safety, adaptability, and empirical confirmation of physiological parameters through imaging and laboratory data. Also, limitations include reliance on case reports, heterogeneous anatomical descriptions, and absence of randomized data due to nature of very rare disease.

## 6 Conclusion

Pharmacologic management in conjoined twins represents a multidisciplinary challenge that demands individualized assessment, diagnostic precision, and careful procedural planning. Safe and effective therapeutic protocols depend on a comprehensive understanding of anatomy, pharmacokinetic variability, and risk mitigation strategies. Clinical pharmacists, working in close collaboration with neonatologists, anesthesiologists, and surgeons, play a pivotal role in tailoring regimens, safeguarding documentation accuracy, and ensuring bedside safety. Looking ahead, future priorities should include the development of PBPK models adapted to conjoined twins’ physiology, the establishment of multicenter registries for prospective data collection, and long-term follow-up studies to capture developmental pharmacology outcomes. The integration of simulation and virtual reality into conjoined twins’ pharmacology education may further strengthen training and preparedness. Ultimately, cautious empiricism, rigorous monitoring, and ethical transparency remain the cornerstones of safe and responsible pharmacotherapy in these rare and complex patients.

## Data Availability

The original contributions presented in the study are included in the article/supplementary material, further inquiries can be directed to the corresponding author.
